# Global prevalence of obesity and overweight among medical students: a systematic review and meta-analysis

**DOI:** 10.1186/s12889-024-19184-4

**Published:** 2024-06-24

**Authors:** Arman Shafiee, Zahra Nakhaee, Razman Arabzadeh Bahri, Mohammad Javad Amini, Amirhossein Salehi, Kyana Jafarabady, Niloofar Seighali, Pegah Rashidian, Hanieh Fathi, Fatemeh Esmaeilpur Abianeh, Samira Parvizi Omran, Mahmood Bakhtiyari, Amirhesam Alirezaei

**Affiliations:** 1 Student Research Committee, School of Medicine, Alborz University of Medical Sciences, Karaj, Iran; 2 Student Research Committee, School of Medicine, Gonabad University of Medical Sciences, Gonabad, Iran; 3 School of Medicine, Tehran University of Medical Sciences, Tehran, Iran; 4 School of Medicine, Shahid Beheshti University of Medical Sciences, Tehran, Iran; 5 School of Medicine, Guilan University of Medical Sciences, Rasht, Iran; 6 Non-Communicable Diseases Research Center, Alborz University of Medical Sciences, Karaj, Iran

**Keywords:** Obesity, Medical students, Overweight, Prevalence

## Abstract

**Background:**

Obesity is a global health concern, and understanding its prevalence among medical students is crucial for shaping targeted interventions. This systematic review and meta-analysis aim to comprehensively assess the prevalence of obesity and overweight among medical students.

**Methods:**

A systematic literature search was conducted across major databases, including PubMed, Scopus, and Web of Science, in order to identify relevant studies that evaluated obesity and overweight among medical students. Inclusion criteria encompassed published and peer-reviewed studies reporting the prevalence of obesity among medical students.

**Results:**

A total of 1245 studies were screened based on their titles and abstracts, and 99 studies comprised a total sample size of 47,455 medical students across diverse geographical regions were included in this study. The overall pooled prevalence of overweight among medical students was estimated at 18% (95% CI: 17%—20%), with obesity at 9% (95% CI: 7%—11%). The combined prevalence of excess weight (overweight and obesity) was calculated to be 24% (95% CI: 22%—27%). Meta-regression results indicated a significant correlation between study year and overweight/obesity prevalence (*p* < 0.05), with a trend towards increasing prevalence over time. Male medical students exhibited a higher pooled prevalence, increasing with the percentage of male participants.

**Conclusion:**

This systematic review and meta-analysis provide a comprehensive overview of the prevalence of obesity among medical students globally. In summary, obesity and overweight present a substantial worldwide health concern, especially among susceptible groups such as medical students, whose prevalence is on the rise. It is crucial to grasp the extent and contributing factors of obesity among medical students to formulate precise interventions aimed at fostering healthier habits and alleviating the adverse impacts of obesity on both physical and mental health.

**Supplementary Information:**

The online version contains supplementary material available at 10.1186/s12889-024-19184-4.

## Introduction

In recent decades, obesity has emerged as a global health concern, and its prevalence is increasing dramatically worldwide [1–3]. Obesity is characterized by excessive accumulation of body fat within adipose tissue, which may lead to adverse health effects [4]. Globally, body mass index (BMI) is the most commonly used to classify overweight and obesity in adults and is defined as weight in kg/height in m^2^. Individuals with a BMI between 25 and 29.9 kg/m^2^ are considered overweight, and Individuals with a BMI ≥ 30kg/m^2^ are considered obese. Obesity is further classified into three severity levels: class I (BMI 30.0–34.9), class II (BMI 35.0–39.9), and class III (BMI ≥ 40.0) [5]. Several studies have identified obesity and overweight as risk factors for chronic and life-threatening illnesses, including diabetes [6], various cancers [7, 8], cardiovascular disease [9], and hypertension [10, 11]. The increasing prevalence of obesity and overweight, and its resulting mortality and morbidity, threaten people’s health in many countries. In addition, it causes destructive health conditions and financial burdens on people and society [12, 13].

Obesity is a multifactorial pathology, and it has been suggested that the increasing prevalence can be attributed to lifestyle changes, particularly nutritional behavior and inadequate physical activity [14–16]. While the general population is affected by the obesity epidemic, certain subgroups, such as medical students, may be particularly vulnerable. Medical students, a population that should act as healthy role models, often face unique challenges that can contribute to unhealthy lifestyle habits, including long hours of studying, high levels of stress, and limited time for physical activity and self-care [17]. Shift work may have significant repercussions on the health of the worker and has been linked to unhealthy lifestyles [18]. A study demonstrated that those who work in shifts have a greater risk of being obese than regular 8-h workers [19]. Furthermore, medical students face a higher risk of developing psychological issues, such as feeding and eating disorders (FEDs) [20–22]. A study estimated that the prevalence of FEDs symptoms in medical students is approximately 17.35% [23]. Socioeconomic and psychological elements significantly affect dietary habits and physical inactivity [23]. Eating habits have a stronger impact on BMI than physical activity [24]. The dietary habits observed among medical students include irregular meals, skipping meals, insufficient intake of fruits and vegetables, high consumption of candies and alcohol, and excessive consumption of fried and fast foods [23, 25, 26]. Accordingly, exposure to these known and unknown factors may increase the risk of overweight and obesity among medical students.

Given the fact that obesity negatively impacts an individual’s physical and mental health [27], understanding the prevalence of obesity among medical students is crucial for identifying potential risk factors and developing targeted interventions to promote healthier lifestyles within this population. Several studies from different countries have reported the prevalence of obesity among medical students [28–30]. However, to the best of our knowledge, this study is the first systematic review and meta-analysis to explore the current state of obesity prevalence among medical students. Also, our study aims to take advantage of all available data on the topic to offer new insights into the prevalence and distribution of obesity within BMI subgroups in medical students.

## Methods

The primary objective of this study is to investigate the prevalence of obesity and overweight among medical students globally. Following the PRISMA (Preferred Reporting Items for Systematic Reviews and Meta-Analyses) checklist [31], our methodology encompasses key steps to ensure transparency and rigor in our research.

### Research question

Our research seeks to ascertain the global prevalence of obesity and overweight among medical students, with a specific focus on studies employing body mass index (BMI) as the primary metric for the measurement of obesity and overweight.

### Search strategy

We conducted a comprehensive search across various databases, including PubMed, Scopus, and Web of Science, from the inception to August 4th, 2023, to identify relevant studies. The search terms included variations of "medical students," "obesity," "overweight," and "BMI."

### Eligibility criteria

The population, intervention, comparison, and outcome (PICO) framework was followed in this study and were as follows: Population (P): medical students; Intervention (I): none; Comparison (C): overweight, obese, or healthy medical students; and Outcome (O): prevalence of obesity or overweight among medical students. We included cross-sectional, descriptive, observational studies conducted globally that involved medical students. Studies were considered if they explored the prevalence of obesity and overweight, using BMI as the measurement tool. We excluded studies that did not meet these criteria or lacked essential information. No limitation was imposed regarding the original language of the identified articles or the gender of the evaluated medical students.

### Study selection

Two independent reviewers screened the identified studies based on the title and abstract. Full-text assessments were performed to ensure the inclusion of relevant data. Any discrepancies in selection were resolved through discussion or consultation with a third reviewer.

### Data extraction

We extracted pertinent information from the selected studies, including study design, geographic location, sample size, and prevalence rates of obesity and overweight among medical students. We prioritized data collected using the World Health Organization (WHO) criteria for obesity and overweight classification (BMI > 30 for obese, 25 < BMI ≤ 30 for overweight).

### Quality assessment

The Newcastle–Ottawa Scale (NOS), which is a validated and easy-to-use scale, was used to assess the quality of the included articles (Supplemental Table 1). The NOS for cross-sectional studies contains seven items within three domains, including selection, comparability, and outcome, with an overall score of nine. The selection domain has four questions and a maximum score of five scores. The comparability domain has a maximum score of one. The outcome domain has two questions and a maximum score of three scores. A score of 7–9 indicates high quality, 4–6 indicates high risk, and 0–3 indicates very high risk of bias. Quality assessment was checked independently by two authors, and any disagreements were resolved by a third author.

### Data synthesis

We synthesized the extracted data using a random effect meta-analysis, synthesizing the overall prevalence rates of obesity and overweight among medical students. Subgroup analyses were conducted based on geographic regions and study characteristics to explore potential variations. Publication bias was examined through doi plots and Peter's test, with statistical relevance set at a *p*-value below 0.1 [32, 33]. All statistical operations and the production of graphs were conducted using STATA and R software(meta package) [34].

## Results

**Fig. 1:**
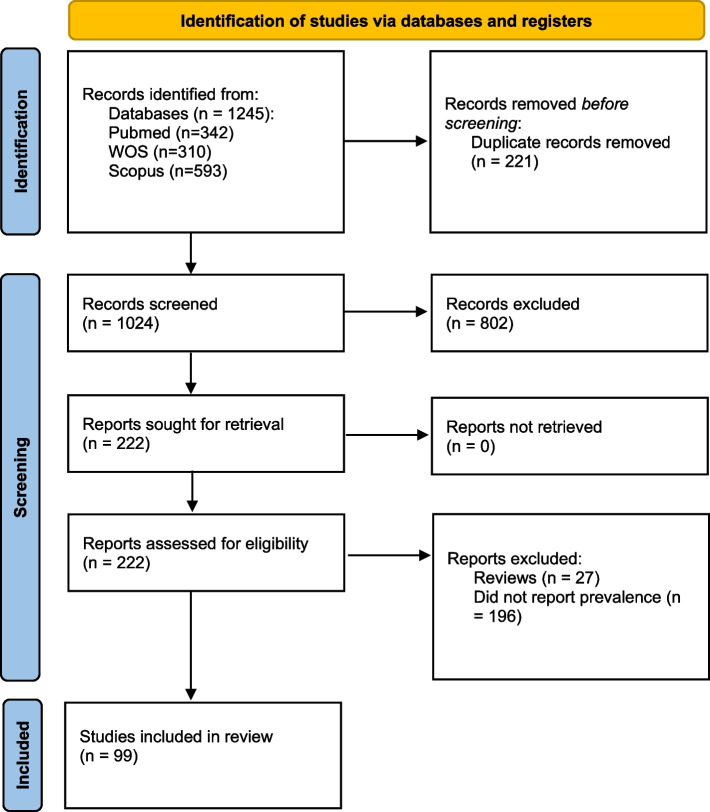
PRISMA flow diagram

The systematic review and meta-analysis aimed to examine the prevalence of overweight, obesity, and overall excess weight among medical students. A comprehensive search of electronic databases identified 1,245 articles. After screening titles and abstracts, 254 articles underwent full-text review, with 99 studies meeting the inclusion criteria and included in the meta-analysis (Fig. 1).

### Characteristics of included studies

The 100 included studies encompassed a total sample size of 47,455 medical students. These studies were conducted across diverse geographical regions, representing both developed and developing countries. The included studies were conducted in Bahrain, Bangladesh, Bosnia and Herzegovina, Cameron, China, Egypt, Saudi Arabia, Greece, India, Iran, Iraq, Lithuania, Malaysia, Mexico, Morocco, Nepal, Oman, Pakistan, Poland, Romania, Russia, Singapore, Slovakia, South Africa, Spain, Sudan, Syria, Thailand, Tunisia, Turkey, United Arab Emirates, the United States of America, and the United Kingdom. Predominantly, cross-sectional designs were employed, and data collection periods ranged from 1992 to 2023. However, most studies were published in recent years, ranging from 2018 to 2023.

### Prevalence of overweight, obesity, and excess weight

**Fig. 2:**
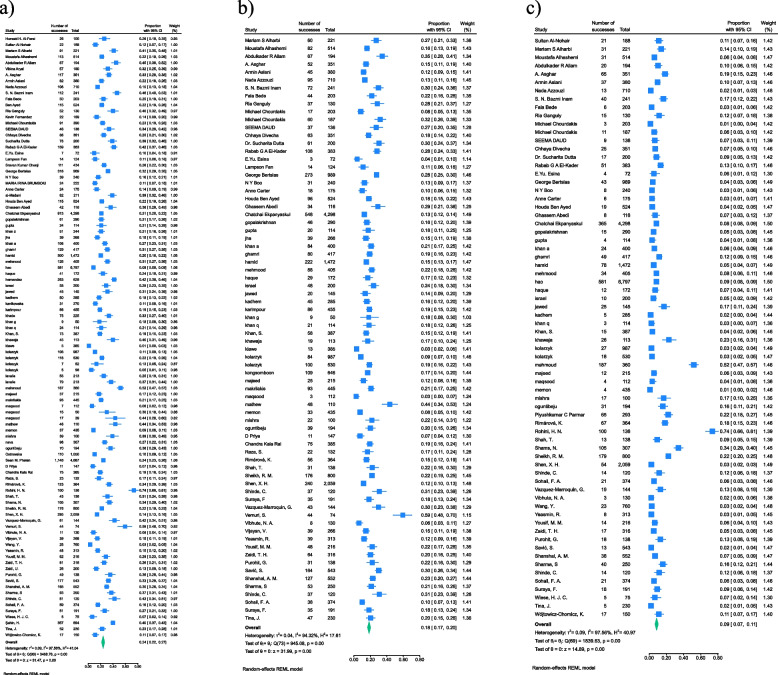
Results of meta-analysis for the prevalence of (**a**) excess weight (overweight/obesity); (**b**) overweight; and (**c**) obesity among medical students

The overall pooled prevalence of overweight among medical students was estimated to be 0.18 (95% CI: 0.17 – 0.20), while the pooled prevalence of obesity was 0.09 (95% CI: 0.07 – 0.11). The combined prevalence of excess weight (overweight and obesity) was calculated to be 0.24 (95% CI: 0.22 – 0.27) (Fig. 2).

### Meta-regression analysis

**Fig. 3:**
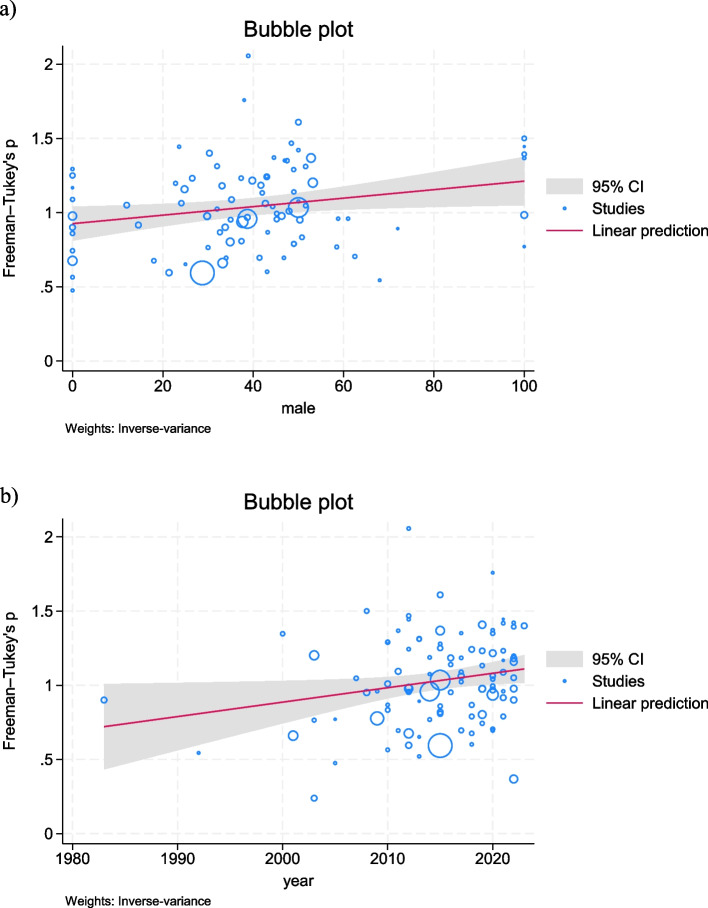
Scatter plot of meta-regression analysis for the association between (**a**) percentage of male participants, and (**b**) study year with the prevalence of overweight/obesity among medical students. Bubble size represents the weight of the study

A meta-regression was conducted to explore potential sources of heterogeneity across studies. Variables such as study year, percentage of male participants, and mean age of population were considered. The results indicated that the study year significantly correlated with overweight/obesity prevalence (*p* < 0.05) (Fig. 3), with a trend towards increasing prevalence over time. Male medical students exhibited a higher pooled prevalence of overweight/obesity, as the prevalence increased with the increased percentage of male participants. No significant associations were observed between the mean age of the population and the aforementioned outcomes (Supplementary Table 2).

### Publication bias

**Fig. 4:**
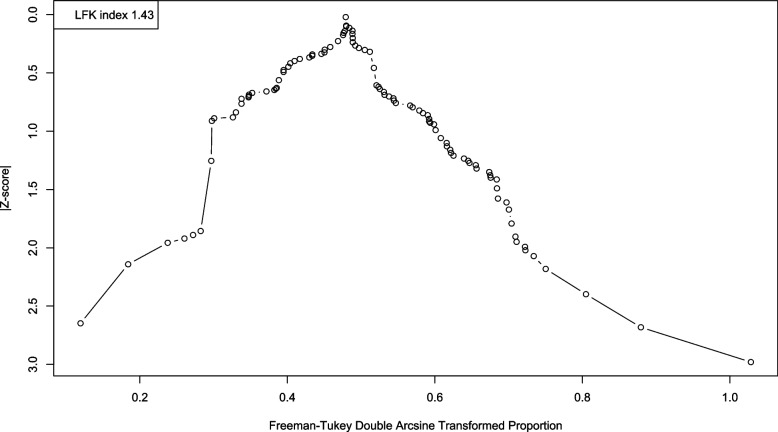
Doi plot for prevalence of excess weight (overweight and obesity) among medical students

Doi plot and Peter’s regression test showed possible publication bias across the included studies for the primary outcome (Fig. 4) (*p*-value < 0.001).

## Discussion

Obesity has become one of the greatest health burdens of our era. As the World Health Organization states, around 2 billion people worldwide were reported to be overweight in 2016, of which more than 650 million people were considered to be obese, something around 13% of the whole population [35]. Globally speaking, 37% of men and 38% of women are considered to be overweight with a BMI greater than 25 kg/m2 [36]. Around 50% of obese people are distributed in only 10 countries, including the United States, China, India, Russia, Brazil, Mexico, Egypt, Germany, Pakistan, and Indonesia. In Europe, there is an upward trend towards obesity, and 17% of adults are obese [37]. As it has been long noticed before, obesity is not only an appearance complication but can also be a risk factor for health conditions of great significance, such as hypertensive diseases, dyslipidemia, obstructive sleep apnea, cancers, and etc. [11, 38, 39]

In the present systematic review and meta-analysis, we aimed to inquire into the prevalence of overweight, obesity, and overall excess weight among medical students. Overall, 254 studies were fully reviewed, of which 99 articles met the inclusion criteria and were used in this study. The sample consisted of 48,683 medical students coming from diverse backgrounds, representing both high and low-income countries. Data extraction was performed on relevant studies since 1992 to 2023. The total pooled prevalence of overweight among medical students was estimated to be 18.5% (95% CI: 16.5%—20.5%), while the pooled prevalence of obesity was 9% (95% CI: 7%—11%). The combined prevalence of excess weight (overweight and obesity) was calculated to be 24% (95% CI: 21%—26%). Moreover, the results specified that there is an obvious association between the year the study was conducted and the prevalence of overweight/obesity, meaning as time passes, the prevalence grows. Furthermore, it was indicated that male medical students had a slightly higher pooled prevalence of overweight/obesity.

Medical education is known to be one of the most demanding academic subjects there are. Education programs are usually too time-consuming, and plenty of medical students tend to ignore the importance of healthy nutrition and physical activity. In a study by Shah T. et al. (2014), 34% of medical students consumed fast food because healthy homemade food was just not available [40]. In another study by Savić S. et al. (2020), a major part of medical students did not have any form of physical activity throughout the week (64.3% of the study population) [41]. It has also been stated in another paper that university students with BMIs in normal ranges tend to participate more regularly in physical activities than underweight or overweight students [42]. Since medical students are the next generation’s medical doctors and, therefore, future leaders of health care procedures, it is of utmost importance to find out if overweight and obesity can be an actual concern for the group.

Throughout the years, there have been a variety of studies focusing on the matter of excessive body weight in medical students. In the present study, we tried to gather such studies and assess and possibly compare their results. In a cross-sectional study by Bazmi Inam, S. N. (2008), overall, 112 out of 241 students (46.5%) in the study were reported to be overweight or obese (BMI > 25) [43]. A different research by Gopalakrishnan S. et al. (2012) showed that of the 169 medical students who participated in the study, respectively 21.3% and 26.6% were discovered to be obese and overweight, of whom above 50% didn’t exercise regularly, 60.4% did not consume the necessary portions of fruits and vegetables daily, and 68% had a positive family history of Diabetes Mellitus [44]. In another cross-sectional descriptive study done by Purohit G. et al. (2015), the prevalence of medical students with a BMI more than 25 in a 138-participiant sample was 35.5%. The study also stated that more than 90% of the participants were consuming fast food [45]. Smrithi Krishnamohan et al. designed a non-randomized controlled trial in a private medical college located in India to measure the efficacy of health education using social networking sites in promoting healthy lifestyles among medical students. The sample was selected from overweight/obese individuals, and all participants were divided into two groups: with (intervention arm) and without a Facebook account (control arm). Results showed a significant decrease in BMI among the control group. They came to the conclusion that except for the decrease in junk food intake, the use of Facebook as an effective tool to promote a healthy lifestyle, e.g., weight reduction, could not be proved confidently [46]. In a study by Bing Li et al., the association between body composition and physical fitness among Chinese medical students was assessed. A total of 2291 medical students were recruited to participate in this cross-sectional study. They concluded that higher fat mass was significantly associated with worse physical fitness among medical students [47]. Miloš Ž. Maksimović et al. carried out a cross-sectional study to assess the knowledge and approach of medical students towards cardiovascular disease (CVD) risk factors, e.g., obesity and overweight. They also compared 2nd year and last year’s medical student’s knowledge regarding the CVD risk factors. Results indicated that last year medical students were significantly more knowledgeable than those at the beginning of their studies. However, their total awareness of such risk factors needs serious improvement [48].

In order to further broaden our view, it is vital to compare the obesity statistics among medical students with those of non-medical students. In a study by Tokaç Er, N. et al. (2021), the overweight and obesity rates amongst 984 undergraduate students from Ankara University Faculty of Health Sciences were respectively 16.5% and 4.5% [49]. Jiang S. et al. (2018) conducted a study to assess the prevalence of overweight and obesity in a sample of 11,673 Chinese college students; results showed a 9.5% rate for overweight and obesity combined [50]. Further analyzing such studies and comparing them to similar studies in medical students might reveal a noticeable difference between the two groups.

The present study has strengths on several sides. First, we followed the PRISMA guidelines to ensure transparency and rigor in our research. Second, our search was as comprehensive as possible. We utilized three major databases (Pubmed, Scopus, and Web of Science) to cover all relevant articles. Third, every included article was quality assessed based on the Newcastle Ottawa assessment tool for cross-sectional studies. Fourth, in the meta-analysis phase, we carried out a subgroup analysis based on geographic regions and study characteristics to find any potential variations. Finally, based on our meta-regression analysis, we found out that as time passes, more medical students are prone to obesity, and also more male students are in danger of excess body weight than the female population.

Despite the mentioned strengths, our study had some noticeable limitations. First, in recent years, the COVID-19 pandemic has seriously affected everyone’s lifestyle and somehow transformed it into a more stressful one. Medical students are no exception in this matter. Thus, more evaluation of the possible impacts of the COVID-19 pandemic on medical students’ weight changes is of great interest. Another thing that could perhaps be classified as a limitation was the lack of nationality diversity among the included studies. Factors like diet and tendency to exercise can be poles apart in different parts of the world. That being so, a more nationally diverse set of studies can aid us in a better assessment of the medical students’ obesity rate. Most of the studies calculated BMI from self-reported weights and heights. It is crucial for studies to indicate the how they measured height and weight so that the actual assessment can be highlighted. Furthermore, we recognize that there can be a tendency in published papers to overrepresent their statistically significant findings. Moreover, the unpublished or grey literature that was not included in this review article can perhaps lead to an incomplete picture of obesity in medical students.

As always, there is room for further research; other groups of students who may be at risk of obesity can be targeted in the future. For instance, the same topic could be assessed among the populations of dental students and medical specialty residents. Furthermore, similar systematic reviews can be conducted to evaluate the prevalence of unhealthy diet and inadequate exercise among medical students as contributing factors for overweight and obesity.

In conclusion, we conducted a systematic review and meta-analysis to assess the prevalence of obesity and overweight among medical students and further understand the significance of obesity among them. Herein, we included a total of 99 articles. The results exhibited that the combined prevalence of excess weight (overweight and obesity) was calculated to be 24%. Owing to the fact that excess body weight can be the leading point of many health problems such as diabetes mellitus, hypertension, psychological disorders, and many more, perhaps counseling medical students to maintain healthier lifestyles can avoid plenty of such health issues [51, 52].

### Supplementary Information

Supplementary Material 1.

## Data Availability

No datasets were generated or analysed during the current study.
