# Supplementary material for: Global prevalence of obesity and overweight among medical students: a systematic review and meta-analysis
Source: BMC Public Health. 2024 Jun 24;24:1673. doi: 10.1186/s12889-024-19184-4 (PMC11194880; doi:10.1186/s12889-024-19184-4)
Supplement: Supplementary file 1 — Supplementary Material 1. [file 12889_2024_19184_MOESM1_ESM.docx]

# Supplementary Material

**Global Prevalence of obesity and overweight among medical students: a systematic review and meta-analysis.**

**Supp Table 1-** Characteristics of included studies.

**Supp Table 2-** Results of meta-regression.

**Supp Table 3-** Results of quality assessment

The authors have provided this supplemental material to give readers additional information about their work.

**Supp Table 1-** Characteristics of included studies.

| Author | Year | Study design | Country | Medical students  (Total/ include) | Age (mean ± SD) | Male  (%) |
| --- | --- | --- | --- | --- | --- | --- |
| Humaid H. Al-Farai [1] | 2014 | cross-sectional | Oman | (-/100) | NR | 50 |
| Sultan Al-Nohair [2] | 2020 | cross-sectional | Saudi Arabia | (200-188) | 22 ± 1.83 | 62.5 |
| Mariam S Alharbi [3] | 2022 | cross-sectional | Saudi Arabia | (-/221) | NR | 100 |
| Moustafa Alhashemi [4] | 2022 | cross-sectional | Syria | (-/514) | NR | 46.3 |
| Abdulkader R Allam [5] | 2012 | cross-sectional | Saudi Arabia | (372-194) | 21.06±1.85 | 48.45 |
| Vibina Aryal [6] | 2022 | cross-sectional | Nepal | (537-180) | NR | NR |
| A. Asghar [7] | 2019 | cross-sectional | India | (4000-351) | 21.34±1.47 | 26.5 |
| Armin Aslani [8] | 2020 | cross-sectional | Iran | 380 | 22.74±3.11 | 38.7 |
| Nada Azzouzi [9] | 2019 | cross-sectional | Morocco | (730-710) | 21.27±2.02 | 34.9 |
| S. N. Bazmi Inam [10] | 2008 | cross-sectional | Saudi Arabia | 241 | 21.2±1.3 | 100 |
| Fala Bede [11] | 2020 | cross-sectional | Cameroon | 203 | 20.8±1.7 | 44.3 |
| Ria Ganguly [12] | 2020 | cross-sectional | India | 130 | 21.99±0.96 | 44.6 |
| Kevin Fernandez [13] | 2014 | cross-sectional | India | (182-159) | F=19±0.76 M=19.12±0.86 | 58.5 |
| Michael Chourdakis [14] | 2010 | cross-sectional | Greece | 390 | M=21.5±1.9 F=21.3±2.2 | 47.94 |
| SEEMA DAUD [15] | 2012 | cross-sectional | Pakistan | 136 |  | 43 |
| Chhaya Divecha [16] | 2022 | cross-sectional | Oman | (500-351) | 21.6±2.2 | 12 |
| Dr. Sucharita Dutta [17] | 2020 | cross-sectional | India | 200 |  | 47.5 |
| Rabab G A El-Kader [18] | 2023 | cross-sectional | United Arab Emirates | (400-383) | 22.75±6.46 | 30.3 |
| E.Yu. Esina [19] | 2013 | cross-sectional | Russia | 72 | 21.9±0.1 | 25 |
| Lampson Fan [20] | 2020 | cross-sectional | UK | (614-124) | 20.6±4 | 46.8 |
| Sravan Kumar Chenji [21] | 2017 | cross-sectional | India | 434 |  | 42.7 |
| George Bertsias [22] | 2003 | cross-sectional | Greece | 989 | 22±2 | 53.2 |
| N Y Boo [23] | 2010 | cross-sectional | Singapore | 240 |  | 50.8 |
| MARIA IRINA BRUMBOIU [24] | 2018 | cross-sectional | Romania | 222 | 21.5±1.9 | 18 |
| Anne Carter [25] | 2003 | cross-sectional | United Arab Emirates | (232-175) | NR | 30 |
| al-Madani, Khawlah M. [26] | 2000 | cross-sectional | Bahrain | 211 | NR | NR |
| Houda Ben Ayed [27] | 2019 | cross-sectional | Tunisia | 524 | 22±3.4 | 29.8 |
| Ghassem Abedi [28] | 2010 | cross-sectional | Iran | 116 |  | 0 |
| Chatchai Ekpanyaskul [29] | 2014 | cross-sectional | Thailand | 5441-4298 | 20.8 ± 2.0 | 38.7 |
| Gopalakrishnan [30] | 2012 | cross-sectional | Malaysia | 290 | 19-25 | 45.2% |
| Gupta [31] | 2009 | cross-sectional | India | 114 | n/a | 61.4% |
| khan z [32] | 2016 | cross-sectional | Pakistan | 244 | 20 | 35% |
| Jha [33] | 2021 | cross-sectional | Nnepal | 266 | 17-25 | 49% |
| khan a [34] | 2011 | cross-sectional | Pakistan | 400 | 18-22 | n/a |
| Ghamri [35] | 2022 | cross-sectional | Saudi Arabia | 417 | 21.65±1.51 | 33.1% |
| Hamid [36] | 2020 | cross-sectional | Pakistan | 1472 | 69.6% were in the age group 20 to 23 | 37.6% |
| Mehmood [37] | 2016 | cross-sectional | Saudi Arabia | 405 | 19-25 | 41.7% |
| Hao [38] | 2015 | cross-sectional | China | 6797 | ??? | 28.74% |
| Haque [39] | 2017 | cross-sectional | Malaysia | 172 | Year-I and II medical students | 32% |
| Hernandez [40] | 2019 | cross-sectional | Spain | 628 | born after 1985 |  |
| Israel [41] | 2016 | Cross-sectional | India | 200 | 18-24 | 49% |
| Jawed [42] | 2017 | cross-sectional | Pakistan | 145 | 18.4 | NR |
| Kadhem [43] | 2018 | cross-sectional | Iraq | 285 | 20 | 32.63% |
| Karimpour [44] | 2022 | cross-sectional | Iran | 455 | 22.31 | 33.8% |
| Khade [45] | 2021 | cross-sectional | India | 225 | (24.9%), belongs to 18 yrs, (52.4%) 19 yrs and (22.6%) belong to 20 yrs (22.6%) | 37.33% |
| khan g [46] | 2013 | cross-sectional | India | 50 | 17-23 | 72% |
| khan q [47] | 2021 | cross-sectional | Pakistan | 114 | n/a | 58.8% |
| Khawaja [48] | 2011 | cross-sectional | Saudi Arabia | 113 | 19.8 | 100% |
| Kini [49] | 2017 | cross-sectional | India | 74 | 17-20 | 45.94% |
| Klawe [50] | 2003 | cross-sectional | Poland | 385 | NR | NR |
| Kolarzyk [51] | 2012 | cross-sectional | Poland/Belarus/Russia/Lithuania | 1517 | 20.9 | 35.06% |
| Kolarzyk [52] | 2005 | cross-sectional | Poland | 150 | 22.2 | 34.66% |
| Kongsomboon [53] | 2010 | cross-sectional | Thailand | 646 | 20.3 | 26.55% |
| Lavalle [54] | 2015 | cross-sectional | Mexico | 213 | 23.26 | 51.6% |
| Mahmoud [55] | 2015 | cross-sectional | Egypt & Saudi Arabia | 360 | 21.43 | 50% |
| Majeed [56] | 2015 | cross-sectional | Saudi Arabia | 215 | 19.27 | 0% |
| Makrilakis [57] | 2008 | cross-sectional | Greece | 445 | 23.2 | 50.33% |
| Maqsood [58] | 2013 | cross-sectional | Pakistan | 112 | NR | NR |
| Mathew [59] | 2012 | cross-sectional | United Arab Emirates | 110 | NR | 23.6% |
| Memon [60] | 2013 | cross-sectional | Pakistan | 73 | 4th year MBBS medical students??? | NR |
| Memon [61] | 2012 | cross-sectional | Pakistan | 435 | 20.5 | 21.37% |
| Mishra [62] | 2021 | cross-sectional | India | 100 | 17-22 | 43% |
| Mishra [63] | 2017 | cross-sectional | India | 100 | 1st year medical students | 47% |
| Mohapatra [64] | 2016 | cross-sectional | India | 200 | 18-20 | 0% |
| Nava [65] | 2021 | cross-sectional | Mexico | 367 | 19.8 | 35.2% |
| Oguntibeju [66] | 2010 | cross-sectional | South Africa | 194 | 25.3 | 48.96% |
| Ostrowska [67] | 2001 | cross-sectional | Poland | 1050 | N.R | 33.23% |
| Piyushkumar C Parmar [68] | 2017 | cross-sectional | India | 293 | 18.65 ± 1.45. | 46% |
| Sean M. Phelan [69] | 2015 | cross-sectional | U.S.A | 5833/ 1146 | 23.9 | 50% |
| D Priya [70] | 2010 | Cross-sectional | India | 147 | 18- x>23 | nr |
| Chandra Kala Rai [71] | 2021 | cross-sectional | Nepal | 385 | 19.95±1.18 | 14.58% |
| Raza, S. [72] | 2010 | cross-sectional | Pakistan | 132 | 20.85 ± 1.21 | 43.20% |
| Rimárová, K. [73] | 2018 | cross-sectional | Slovakia | 364 | 23.47 | 43% |
| Rohini, H. N. [74] | 2012 | cross-sectional | India | 148/136 | boys: 19.1±0.82 girls:18.9± 0.92 | 38.90% |
| Shah, T. [75] | 2014 | cross-sectional | U.S.A | 147/138 | teenagers and youth (nr) | nr |
| Shams, N. [76] | 2015 | cross-sectional | United Arab Emirates | 307 | 22 ± 1.36 | 0% |
| Sheikh, R. M. [77] | 2012 | cross-sectional | Pakistan | 800 | 17-25 | 0% |
| Shen, X. H. [78] | 2009 | cross-sectional | China | 2251/2059 | 21.5 | nr |
| Skemiene, L. [79] | 2007 | cross-sectional | Lithuania | 335 | 18 and 20 years old | 29% |
| Shinde, C. [80] | 2022 | cross-sectional | India | 120 | 21.11± 1.45 | 50% |
| Sohail, F. A. [81] | 2015 | cross-sectional | Pakistan | 412/374 | nr | nr |
| Suraya, F [82] | 2017 | cross-sectional | Saudi Arabia | 191 | 21.31 | nr |
| Vazquez-Marroquin, G. [83] | 2021 | cross-sectional | Mexico | 144 | 22.2 ± 1.7 | nr |
| Vemuri, S. [84] | 2020 | cross-sectional | India | 100/74 | 18-24 | 38.00% |
| Vibhute, N. A. [85] | 2018 | cross-sectional | India | 130 | nr | 43.07% |
| Vijayan, V. [86] | 2018 | cross-sectional | Nepal | 266 | 17-25 | 49% |
| Wang, Y. [87] | 2022 | cross-sectional | China | 760 | nr | nr |
| Yeasmin, R. [88] | 2015 | cross-sectional | Bangladesh | 313 | 20.12 | 37.40% |
| Yousif, M. M. [89] | 2019 | cross-sectional | Sudan | 216 | 19.99 ± 1.86 years | 42% |
| Zaidi, T. H. [90] | 2020 | cross-sectional | Pakistan | 316 | 21.48±4.08 years | 24.10% |
| Zaidi, U. [91] | 2019 | cross-sectional | Saudi Arabia | 400/200 | 21.05 | 0% |
| Purohit, G. [92] | 2015 | cross-sectional | India | 138 | 21.5 |  |
| Savić, S. [93] | 2020 | cross-sectional | Bosnia and Herzegovina | 601/543 | 18-29 | 39.80% |
| Shanshal, A. M. [94] | 2022 | cross-sectional | Iraq | 552 | 22-25 | 24.80% |
| Sharma, S [95] | 2013 | cross-sectional | India | 350/250 | 18-25(21.5) | 32% |
| Wiese, H. J. C. [96] | 1992 | cross-sectional | U.S.A | 75 | 23.7 ± 2.1 | 68% |
| Şahin, H. [97] | 2015 | cross-sectional | Turkey | 849 | 21.37 | 52.80% |
| Tina, J. [98] | 2020 | cross-sectional | India | 230 | 17-24 | 45.20% |
| Wójtowicz-Chomicz, K. [99] | 2011 | cross-sectional | Poland | 150 | 22 | 34% |

**Supp Table 2-** Results of meta-regression.


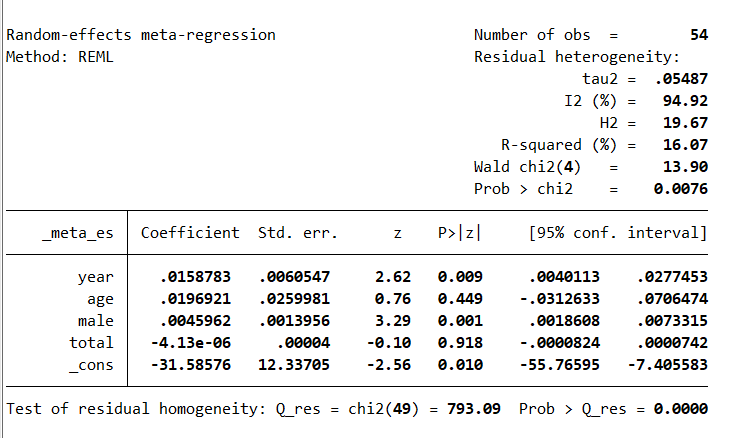


**Supp Table 3-** Results of quality assessment

| ID | Author/Year | 1 | 2 | 3 | 4(max 2 scores) | 5 | 6(max 2 scores) | 7 | Total |
| --- | --- | --- | --- | --- | --- | --- | --- | --- | --- |
| 1 | Humaid H. Al-Farai,2014 | 1 | 0 | 1 | 2 | 0 | 2 | 1 | 7 |
| 2 | Sultan Al-Nohair 2020 | 1 | 1 | 1 | 2 | 1 | 2 | 1 | 9 |
| 3 | Mariam S Alharbi 2022 | 0 | 1 | 1 | 2 | 0 | 2 | 1 | 7 |
| 4 | Moustafa Alhashemi | 1 | 1 | 1 | 2 | 1 | 2 | 1 | 9 |
| 5 | Abdulkader R Allam 2012 | 1 | 1 | 1 | 2 | 1 | 2 | 1 | 9 |
| 6 | Vibina Aryal 2022 | 1 | 1 | 1 | 1 | 0 | 1 | 1 | 6 |
| 7 | A. Asghar 2019 | 1 | 1 | 1 | 1 | 1 | 2 | 1 | 8 |
| 8 | Armin Aslani 2020 | 1 | 1 | 1 | 1 | 1 | 2 | 1 | 8 |
| 9 | Nada Azzouzi 2019 | 1 | 1 | 1 | 1 | 1 | 1 | 1 | 7 |
| 10 | S. N. Bazmi Inam 2008 | 1 | 0 | 1 | 2 | 0 | 1 | 1 | 6 |
| 11 | Fala Bede 2020 | 1 | 0 | 1 | 2 | 0 | 1 | 1 | 6 |
| 12 | Ria Ganguly 2020 | 1 | 0 | 1 | 2 | 0 | 2 | 1 | 7 |
| 13 | Kevin Fernandez 2014 | 1 | 1 | 0 | 2 | 0 | 1 | 1 | 6 |
| 14 | Michael Chourdakis 2010 | 1 | 0 | 1 | 2 | 1 | 1 | 1 | 7 |
| 15 | SEEMA DAUD 2012 | 0 | 0 | 1 | 2 | 0 | 1 | 1 | 5 |
| 16 | Chhaya Divecha 2022 | 1 | 0 | 1 | 2 | 1 | 1 | 1 | 7 |
| 17 | Dr. Sucharita Dutta 2020 | 1 | 0 | 1 | 2 | 1 | 2 | 1 | 8 |
| 18 | Rabab G A El-Kader 2023 | 1 | 0 | 1 | 2 | 1 | 1 | 1 | 7 |
| 19 | E.Yu. Esina 2013 | 0 | 0 | 1 | 2 | 0 | 1 | 1 | 5 |
| 20 | Lampson Fan2020 | 1 | 0 | 1 | 2 | 1 | 2 | 1 | 8 |
| 21 | Sravan Kumar Chenji 2017 | 1 | 1 | 1 | 2 | 1 | 2 | 1 | 9 |
| 22 | George Bertsias 2003 | 1 | 1 | 1 | 2 | 1 | 2 | 1 | 9 |
| 23 | N Y Boo 2010 | 1 | 0 | 1 | 2 | 1 | 2 | 1 | 8 |
| 24 | MARIA IRINA BRUMBOIU 2018 | 1 | 0 | 1 | 2 | 1 | 2 | 1 | 8 |
| 25 | Anne Carter  2003 | 1 | 0 | 0 | 2 | 0 | 2 | 1 | 6 |
| 26 | al-Madani, Khawlah M.2000 | 0 | 0 | 1 | 2 | 0 | 1 | 1 | 5 |
| 27 | Houda Ben Ayed 2019 | 1 | 1 | 1 | 2 | 1 | 1 | 1 | 8 |
| 28 | Ghassem Abedi 2010 | 0 | 0 | 1 | 2 | 1 | 2 | 1 | 7 |
| 29 | Chatchai Ekpanyaskul 2014 | 1 | 1 | 1 | 2 | 1 | 2 | 1 | 9 |
| 30 | Ghamri 2022 | 1 | 0 | 0 | 2 | 0 | 2 | 1 | 6 |
| 31 | Gopalakrishnan 2012 | 1 | 0 | 0 | 2 | 1 | 2 | 1 | 7 |
| 32 | Gupta 2009 | 1 | 0 | 0 | 2 | 0 | 2 | 1 | 6 |
| 33 | Hamid 2020 | 1 | 1 | 1 | 2 | 1 | 2 | 1 | 9 |
| 34 | Hao 2015 | 1 | 1 | 0 | 2 | 1 | 1 | 1 | 7 |
| 35 | Haque 2017 | 1 | 0 | 0 | 1 | 1 | 1 | 1 | 7 |
| 36 | Hernandez 2019 |  | 1 |  |  |  |  |  | 1 |
| 37 | Israel 2016 | 1 | 0 | 0 | 2 | 1 | 1 | 1 | 6 |
| 38 | Jawed 2017 |  | 0 |  |  |  |  |  | 0 |
| 39 | Jha 2021 | 1 | 0 | 1 | 2 | 1 | 2 | 1 | 8 |
| 40 | Kadhem 2018 | 1 | 0 | 0 | 2 | 1 | 1 | 1 | 6 |
| 41 | Karimpour 2022 | 1 | 1 | 0 | 2 | 1 | 2 | 1 | 8 |
| 42 | Khade 2021 | 1 | 0 | 0 | 2 | 1 | 2 | 1 | 7 |
| 43 | khan a 2011 | 1 | 1 | 0 | 2 | 1 | 2 | 1 | 8 |
| 44 | khan g 2013 | 1 | 0 | 0 | 1 | 1 | 0 | 0 | 3 |
| 45 | khan q 2021 | 1 | 0 | 0 | 2 | 1 | 2 | 1 | 7 |
| 46 | khan z 2016 | 1 | 0 | 0 | 2 | 0 | 2 | 1 | 6 |
| 47 | Khawaja 2011 | 1 | 0 | 0 | 2 | 1 | 2 | 1 | 7 |
| 48 | Kini 2017 | 1 | 0 | 0 | 1 | 1 | 2 | 1 | 6 |
| 49 | Klawe 2003 | 1 | 0 | 0 | 1 | 1 | 1 | 1 | 5 |
| 50 | Kolarzyk 2012 | 1 | 1 | 0 | 1 | 0 | 1 | 1 | 5 |
| 51 | Kolarzyk 2005 | 1 | 0 | 0 | 2 | 1 | 2 | 1 | 7 |
| 52 | Kongsomboon 2010 | 1 | 1 | 1 | 2 | 1 | 2 | 1 | 9 |
| 53 | Lavalle 2015 | 1 | 0 | 1 | 1 | 0 | 1 | 1 | 5 |
| 54 | Mahmoud 2015 | 1 | 0 | 0 | 2 | 1 | 2 | 1 | 7 |
| 55 | Majeed 2015 | 1 | 0 | 0 | 2 | 1 | 2 | 1 | 7 |
| 56 | Makrilakis 2008 | 1 | 1 | 0 | 2 | 0 | 2 | 1 | 7 |
| 57 | Maqsood 2013 | 1 | 0 | 1 | 1 | 1 | 1 | 1 | 5 |
| 58 | Mathew 2012 | 1 | 0 | 0 | 2 | 1 | 1 | 1 | 6 |
| 59 | Mehmood 2016 | 1 | 1 | 0 | 2 | 1 | 2 | 1 | 8 |
| 60 | Memon 2013 | 1 | 0 | 1 | 1 | 1 | 1 | 1 | 6 |
| 61 | Memon 2012 | 1 | 1 | 0 | 2 | 1 | 2 | 1 | 8 |
| 62 | Mishra 2021 | 1 | 0 | 0 | 0 | 1 | 1 | 1 | 4 |
| 63 | Mishra 2017 | 1 | 0 | 0 | 2 | 1 | 1 | 1 | 6 |
| 64 | Mohapatra 2016 | 1 | 0 | 0 | 2 | 1 | 1 | 1 | 6 |
| 65 | Nava 2021 | 1 | 0 | ? | 2 | 1 | 1 | 1 | 6 |
| 66 | Oguntibeju 2010 | 1 | 0 | 0 | 2 | 1 | 1 | 1 | 6 |
| 67 | Ostrowska 2001 |  | 1 |  |  |  |  |  | 1 |
| 68 | Piyushkumar C Parmar 2017 | 1 | 0 | 1 | 2 | 1 | 2 | 1 | 8 |
| 69 | Sean M. Phelan 2015 | 1 | 1 | 1 | 2 | 0 | 2 | 1 | 8 |
| 70 | D Priya 2010 | 1 | 0 | 1 | 2 | 0 | 2 | 1 | 7 |
| 71 | Chandra Kala Rai 2021 | 1 | 1 | 1 | 2 | 1 | 2 | 1 | 9 |
| 72 | Raza, S 2010 | 0 | 0 | 1 | 1 | 1 | 2 | 1 | 6 |
| 73 | Rimárová, K. 2018 | 1 | 0 | 1 | 1 | 0 | 2 | 1 | 6 |
| 74 | Rohini, H. N 2012 | 1 | 1 | 1 | 1 | 0 | 1 | 1 | 6 |
| 75 | Shams, N. 2015 | 0 | 0 | 1 | 1 | 0 | 2 | 1 | 5 |
| 76 | Sheikh, R. M. 2012 | 1 | 1 | 1 | 2 | 0 | 2 | 1 | 8 |
| 77 | Shen, X. H. 2009 | 0 | 1 | 1 | 2 | 0 | 1 | 1 | 6 |
| 78 | Skemiene, L. 2007 | 1 | 0 | 1 | 2 | 1 | 2 | 1 | 8 |
| 79 | Shinde, C. 2022 | 1 | 0 | 1 | 2 | 0 | 2 | 1 | 7 |
| 80 | Skemiene, L. 2007 | 1 | 0 | 0 | 2 | 1 | 2 | 1 | 7 |
| 81 | Sohail, F. A. 2015 | 1 | 0 | 1 | 2 | 1 | 2 | 1 | 8 |
| 82 | Suraya, F 2017 | 1 | 0 | 1 | 2 | 0 | 2 | 1 | 7 |
| 83 | Vazquez-Marroquin, G. 2021 | 1 | 1 | 0 | 1 | 0 | 2 | 1 | 6 |
| 84 | Vemuri, S. 2020 | 1 | 0 | 0 | 2 | 0 | 2 | 1 | 6 |
| 85 | Vibhute, N. A. 2018 | 1 | 0 | 0 | 2 | 0 | 2 | 1 | 6 |
| 86 | Vijayan, V. 2018 | 1 | 0 | 2 | 1 | 2 | 2 | 1 | 9 |
| 87 | Wang, Y. 2022 | 1 | 1 | 1 | 2 | 1 | 2 | 1 | 9 |
| 88 | Yeasmin, R. 2015 | 0 | 0 | 1 | 2 | 0 | 2 | 1 | 6 |
| 89 | Yousif, M. M. 2019 | 1 | 1 | 0 | 2 | 1 | 2 | 1 | 8 |
| 90 | Zaidi, T. H. 2020 | 1 | 0 | 1 | 2 | 0 | 2 | 1 | 7 |
| 91 | Zaidi, U. 2019 | 1 | 1 | 1 | 2 | 1 | 2 | 1 | 9 |
| 92 | Purohit, G. 2015 | 1 | 0 | 1 | 1 | 1 | 2 | 1 | 7 |
| 93 | Savić, S. 2020 | 0 | 0 | 1 | 1 | 1 | 2 | 1 | 6 |
| 94 | Shah, T. 2014 | 1 | 1 | 0 | 2 | 1 | 2 | 1 | 8 |
| 95 | Shanshal, A. M. 2022 | 1 | 1 | 1 | 2 | 1 | 2 | 1 | 9 |
| 96 | Sharma, S 2013 | 1 | 0 | 1 | 2 | 1 | 2 | 1 | 8 |
| 97 | Wiese, H. J. C. 1992 | 1 | 1 | 2 | 1 | 1 | 2 | 1 | 9 |
| 98 | Şahin, H. 2015 | 1 | 1 | 1 | 2 | 1 | 2 | 1 | 9 |
| 99 | Tina, J. 2020 | 1 | 0 | 1 | 2 | 1 | 2 | 1 | 8 |

1. Al-Farai, H.H., et al., *Insulin resistance and its correlation with risk factors for developing diabetes mellitus in 100 omani medical students.* Sultan Qaboos Univ Med J, 2014. **14**(3): p. e393-6.

2. Al-Nohair, S., et al., *Cross-sectional study of cardiovascular risk factors among male and female medical students in qassim university – college of medicine saudi arabia.* Open Access Macedonian Journal of Medical Sciences, 2020. **8**(E): p. 439-445.

3. Alharbi, M.S., et al., *Prevalance of overweight, obesity and diabetes in undergraduate medical students at Qassim University.* Revista Latinoamericana de Hipertension, 2022. **17**(6): p. 388-395.

4. Alhashemi, M., et al., *Prevalence of obesity and its association with fast-food consumption and physical activity: A cross-sectional study and review of medical students' obesity rate.* Ann Med Surg (Lond), 2022. **79**: p. 104007.

5. Allam, A.R., et al., *Nutritional and health status of medical students at a university in Northwestern Saudi Arabia.* Saudi Med J, 2012. **33**(12): p. 1296-303.

6. Aryal, V., et al., *Obesity among Medical Students of a Medical College: A Descriptive Cross-sectional Study.* JNMA J Nepal Med Assoc, 2022. **60**(255): p. 943-946.

7. Asghar, A., et al., *Frequency of Pre-obesity and Obesity in Medical Students of Karachi and the Predisposing Lifestyle Habits.* Cureus, 2019. **11**(1): p. e3948.

8. Aslani, A., et al., *Prevalence of obesity and association between body mass index and different aspects of lifestyle in medical sciences students: A cross-sectional study.* Nursing Open, 2021. **8**(1): p. 372-379.

9. Azzouzi, N., et al., *Eating disorders among Moroccan medical students: cognition and behavior.* Psychol Res Behav Manag, 2019. **12**: p. 129-135.

10. Bazmi Inam, S.N., *Prevalence of overweight and obesity among students of a medical college in Saudi Arabia.* Journal of the Liaquat University of Medical and Health Sciences, 2008. **7**(1): p. 41-43.

11. Bede, F., et al., *Dietary habits and nutritional status of medical school students: The case of three state universities in cameroon.* Pan African Medical Journal, 2020. **35**.

12. Ganguly, R., et al., *Physical activity and dietary habits among mbbs students of a private medical college of eastern india.* International Journal of Current Research and Review, 2020. **12**(21): p. 69-75.

13. Fernandez, K., et al., *Study regarding overweight/obesity among medical students of a teaching hospital in Pune, India.* Medical Journal of Dr. D.Y. Patil University, 2014. **7**(3): p. 279-283.

14. Chourdakis, M., et al., *Eating habits, health attitudes and obesity indices among medical students in northern Greece.* Appetite, 2010. **55**(3): p. 722-5.

15. Daud, S. and F. Javaid, *Estimation of body mass index (BMI) in medical students.* Pakistan Journal of Medical and Health Sciences, 2011. **5**(4): p. 702-705.

16. Divecha, C.A. and M.A. Simon, *Body Image Concerns and Weight Control Behaviors among Medical Students in Oman.* Journal of Nature and Science of Medicine, 2022. **5**(1): p. 29-35.

17. Dutta, S., et al., *Study of obesity and hypertension among 1st year MBBS students admitted to Rural Medical College, Loni, Maharashtra.* Pravara Medical Review, 2020. **12**(3): p. 54-60.

18. El-Kader, R.G.A., et al., *Assessment of health-related behaviors among medical students: A cross-sectional study.* Health Science Reports, 2023. **6**(6).

19. Esina, E.Y., A.A. Zuykova, and O.N. Krasnorutskaya, *Cardiovascular risk factors and selected parameters of ECG dispersion mapping in medical students.* Cardiovascular Therapy and Prevention (Russian Federation), 2013. **12**(2): p. 70-73.

20. Fan, L.M., et al., *Impact of unhealthy lifestyle on cardiorespiratory fitness and heart rate recovery of medical science students.* BMC Public Health, 2020. **20**(1).

21. Chenji, S.K., et al., *Cross-sectional analysis of obesity and high blood pressure among undergraduate students of a university medical college in South India.* Family Medicine and Community Health, 2018. **6**(2): p. 63-69.

22. Bertsias, G., et al., *Overweight and obesity in relation to cardiovascular disease risk factors among medical students in Crete, Greece.* BMC Public Health, 2003. **3**: p. 3.

23. Boo, N.Y., et al., *The prevalence of obesity among clinical students in a Malaysian medical school.* Singapore Medical Journal, 2010. **51**(2): p. 126-132.

24. Brumboiu, M.I., et al., *Nutritional status and eating disorders among medical students from the Cluj-Napoca University centre.* Clujul Med, 2018. **91**(4): p. 414-421.

25. Carter, A.O., et al., *Health and lifestyle needs assessment of medical students in the United Arab Emirates.* Med Teach, 2003. **25**(5): p. 492-6.

26. Al-Madani, K.M., *Obesity among medical practitioners and medical students in Bahrain.* Bahrain Medical Bulletin, 2000. **22**(3): p. 138-139.

27. Ben Ayed, H., et al., *Prevalence, determinants and outcomes of general and abdominal obesity in medical students.* Obesity Medicine, 2019. **13**: p. 39-44.

28. Abedi, G., et al., *Study of consumption pattern of food and obesity of female students of Mazandaran University of medical sciences.* Journal of Mazandaran University of Medical Sciences, 2011. **20**(80): p. 76-80.

29. Ekpanyaskul, C., P. Sithisarankul, and S. Wattanasirichaigoon, *Overweight/Obesity and related factors among thai medical students.* Asia Pac J Public Health, 2013. **25**(2): p. 170-80.

30. Gopalakrishnan, S., et al., Med J Malaysia, 2012. **67**(4): p. 442-4.

31. Gupta, S., T.G. Ray, and I. Saha, *Overweight, obesity and influence of stress on body weight among undergraduate medical students.* Indian J Community Med, 2009. **34**(3): p. 255-7.

32. Khan, Z.N., et al., *High prevalence of preobesity and obesity among medical students of Lahore and its relation with dietary habits and physical activity.* Indian J Endocrinol Metab, 2016. **20**(2): p. 206-10.

33. Jha, R.K., et al., *Study of Body Mass Index among Medical Students of a Medical College in Nepal: A Descriptive Cross-sectional Study.* JNMA J Nepal Med Assoc, 2021. **59**(235): p. 280-283.

34. Khan, A.N., et al., *Prevalence of abnormal body mass index among students of a private sector medical College in Pakistan.* Medical Forum Monthly, 2011. **22**(7): p. 57-60.

35. Ghamri, R.A., et al., *Prevalence and predictors of eating disorders: A cross-sectional survey of medical students at King Abdul-Aziz University, Jeddah.* Pak J Med Sci, 2022. **38**(6): p. 1633-1638.

36. Hamid, N., et al., *Variation of BMI in medical students of sialkot.* Medical Forum Monthly, 2020. **31**(1).

37. Mehmood, Y., F.K. Al-Swailmi, and S.A. Al-Enazi, *Frequency of obesity and comorbidities in medical students.* Pak J Med Sci, 2016. **32**(6): p. 1528-1532.

38. Hao, W., et al., *Gender comparisons of physical fitness indexes in Inner Mongolia medical students in China.* Global journal of health science, 2015. **7**(1): p. 220-227.

39. Haque, A.T.M.E., et al., *Relationship between BMI with the anthropometric measurements and the eating habits of the preclinical medical students of Universiti Kuala Lumpur Royal College of Medicine Perak (UniKL RCMP), Malaysia.* Journal of Global Pharma Technology, 2017. **9**(12): p. 1-09.

40. Hernández, A.O., et al., *Association between sleep deficiency and overweight and obesity in new generation medical students in Mexico: A paradigm shift.* Revista Espanola de Nutricion Comunitaria, 2019. **25**(4): p. 152-156.

41. Israel, M., et al., *Obesity in Medical Students and its Correlation with Sleep Patterns and Sleep Duration.* Indian J Physiol Pharmacol, 2016. **60**(1): p. 38-44.

42. Jawed, S., S. Zia, and S. Tariq, *Frequency of different blood groups and its association with BMI and blood pressure among the female medical students of Faisalabad.* J Pak Med Assoc, 2017. **67**(8): p. 1132-1137.

43. Kadhem, Q.I., N. Ghosson Kadhom, and A.A. Jabbar, *Nutritional awareness, dietary rule and fit lifeway of medical students in al Muthanna University.* Journal of Pharmaceutical Sciences and Research, 2018. **10**(7): p. 1672-1676.

44. Karimpour, F.F. and S. Afroughi, *Prevalence of weight status and associated factors of underweight among the medical students in Iran.* New Armenian Medical Journal, 2022. **16**(2): p. 91-99.

45. Khade, Y., et al., *Does body mass index influence cognitive functions among young medical students?* Clinical Epidemiology and Global Health, 2021. **12**.

46. Khan, G.A.N., et al., *Effect of exercise on various blood pressure parameters in different groups of body mass index among young medical students.* Indian Journal of Public Health Research and Development, 2013. **4**(1): p. 93-97.

47. Khan, Q.U., et al., *Frequency of ABO blood groups and its relationship with body mass index in students of a medical college in lahore.* Pakistan Journal of Medical and Health Sciences, 2021. **15**(1): p. 152-156.

48. Khawaja, R.A., S.T. Shaikh, and M.M. Sharif, *Impact of overweight and obesity on ventilatory function among male medical students.* Pakistan Journal of Medical Sciences, 2011. **27**(3): p. 505-509.

49. Kini, S.G. and P.D. Wani, *Correlation between body mass index and response to cold pressor test in young medical students in a tertiary care hospital.* National Journal of Physiology, Pharmacy and Pharmacology, 2017. **7**(3): p. 255-258.

50. Klawe, J.J., et al., *Eating disorders among students of the Medical University in Bydgoszcz.* Przegla̧d lekarski, 2003. **60 Suppl 6**: p. 40-42.

51. Kolarzyk, E., et al., *Nutritional status and food choices among frst year medical students.* Central European Journal of Medicine, 2012. **7**(3): p. 396-408.

52. Kolarzyk, E., A. Ostachowska-Gasior, and A. Skop, *The protein participation in daily diet and nutritional status of medical students in Kraków.* Rocz Akad Med Bialymst, 2005. **50 Suppl 1**: p. 39-41.

53. Kongsomboon, K., *Psychological problems and overweight in medical students compared to students from Faculty of Humanities, Srinakharinwirot University, Thailand.* J Med Assoc Thai, 2010. **93 Suppl 2**: p. S106-13.

54. Lavalle, F.J., et al., *Change in the prevalence of metabolic syndrome in a population of medical students: 6-year follow-up.* J Diabetes Metab Disord, 2015. **14**: p. 85.

55. Mahmoud, A.E.D.H., *Prevalence of cardiovascular disease risk factors among Egyptian and Saudi medical students: A comparative study.* Journal of the Egyptian Public Health Association, 2015. **90**(1): p. 35-39.

56. Majeed, F., *Association of BMI with diet and physical activity of female medical students at the University of Dammam, Kingdom of Saudi Arabia.* Journal of Taibah University Medical Sciences, 2015. **10**(2): p. 188-196.

57. Makrilakis, K., et al., *Correlation of family history of obesity and diabetes mellitus with the BMI of Greek medical students.* Nutr Metab Cardiovasc Dis, 2008. **18**(3): p. e7-8.

58. Maqsood, S.M., et al., *Body mass index (BMI) of international medical Student in OJHA Campus Karachi, Pakistan.* Medical Forum Monthly, 2013. **24**(7): p. 47-50.

59. Mathew, E., et al., *Self-rated health, BMI, blood pressure, and perceived health needs of first year students at a Middle-Eastern medical university.* Preventive Medicine, 2012. **54**(3-4): p. 287.

60. Memon, M.N., A.H. Memon, and A. Abro, *Eating habits and body mass index of medical students of Shaheed Mohtarma Benazir Bhutto Medical University, Larkana.* Medical Forum Monthly, 2013. **24**(6): p. 39-42.

61. Memon, A.A., et al., *Eating disorders in medical students of Karachi, Pakistan-a cross-sectional study.* BMC Research Notes, 2012. **5**.

62. Mishra, T., et al., *Association between Sleep Duration, Use of Electronic Devices and Obesity in Medical Students.* Universal Journal of Public Health, 2021. **9**(6): p. 454-459.

63. Mishra, T., et al., *Association between short sleep duration and obesity in medical students.* Asian Journal of Pharmaceutical and Clinical Research, 2017. **10**(1): p. 242-244.

64. Mohapatra, D., et al., *A study of relation between body mass index and dysmenorrhea and its impact on daily activities of medical students.* Asian Journal of Pharmaceutical and Clinical Research, 2016. **9**: p. 297-299.

65. Nava, E.Y.E., et al., *Body fat percentage associated with diet quality and alcohol consumption in medical students at a public university in Mexico.* Revista Espanola de Nutricion Comunitaria, 2021. **27**(2).

66. Oguntibeju, O.O., R.O. Orisatoki, and E.J. Truter, *The relationship between body mass index and physical activities among medical students in Saint Lucia.* Pakistan Journal of Medical Sciences, 2010. **26**(4): p. 827-831.

67. Ostrowska, L., D. Czapska, and J. Karczewski, *Evaluation of protein, fat and carbohydrate contents in daily dietary allowance of overweight and obese students from the Medical Academy in Białystok.* Roczniki Panstwowego Zakladu Higieny, 2001. **52**(3): p. 247-256.

68. Parmar, P.C., et al., *Exploring the dietary habit of medical students’ and their perception about its effect on health.* Indian Journal of Public Health Research and Development, 2017. **8**(1): p. 154-158.

69. Phelan, S.M., et al., *The Adverse Effect of Weight Stigma on the Well-Being of Medical Students with Overweight or Obesity: Findings from a National Survey.* J Gen Intern Med, 2015. **30**(9): p. 1251-8.

70. Priya, D., et al., *Body Image Perception and Attempts to Change Weight among Female Medical Students at Mangalore.* Indian J Community Med, 2010. **35**(2): p. 316-20.

71. Rai, C.K. and S. Makaju, *Overweight among Medical Students in a Tertiary Care Center: A Descriptive Cross-sectional Study.* JNMA J Nepal Med Assoc, 2021. **59**(240): p. 749-751.

72. Raza, S., et al., *Dietary modification, body mass index (BMI), blood pressure (BP) and cardiovascular risk in medical students of a government medical college of Karachi.* J Pak Med Assoc, 2010. **60**(11): p. 970-4.

73. Rimárová, K., et al., *Prevalence of lifestyle and cardiovascular risk factors in a group of medical students.* Cent Eur J Public Health, 2018. **26 Suppl**: p. S12-s18.

74. Rohini, H.N., P. Kudachi, and S. Goudar, *Association of overnutritional status with anxiety in medical students.* National Journal of Physiology, Pharmacy and Pharmacology, 2012. **2**(2): p. 123-127.

75. Shah, T., et al., *Assessment of obesity, overweight and its association with the fast food consumption in medical students.* J Clin Diagn Res, 2014. **8**(5): p. Cc05-7.

76. Shams, N., et al., *Obesity and hypertension in female medical students; frequency and risk factors.* Journal of the Liaquat University of Medical and Health Sciences, 2015. **14**(1): p. 26-32.

77. Sheikh, R.M., et al., *Relationship of breakfast taking practices and weight among female students of medical colleges of Lahore.* Pakistan Journal of Medical and Health Sciences, 2012. **6**(2): p. 343-347.

78. Shen, X.H., et al., *Body mass index and influencing factors in students of a medical college in Shanghai.* Journal of Shanghai Jiaotong University (Medical Science), 2009. **29**(9): p. 1107-1110.

79. Skemiene, L., et al., *Peculiarities of medical students' nutrition.* Medicina (Kaunas), 2007. **43**(2): p. 145-52.

80. Shinde, C., et al., *A cross-sectional study of obesity related indices and its correlation with blood pressure in Medical students.* Pravara Medical Review, 2022. **14**(3): p. 13-19.

81. Sohail, F.A., Z. Saeed, and B. Bilal, *A cross sectional survey to determine the effect of consuming regular breakfast on BMI in medical students of Jinnah Medical and Dental College.* Medical Forum Monthly, 2015. **26**(8): p. 22-26.

82. Suraya, F., et al., *Effect of obesity on academic grades among Saudi female medical students at College of Medicine, King Saud University: Pilot study.* J Pak Med Assoc, 2017. **67**(8): p. 1266-1269.

83. Vazquez-Marroquin, G., et al., *Dietary intake and anthropometric indices in Mexican medical students, stratified by family history of Type 2 Diabetes.* Revista Espanola de Nutricion Humana y Dietetica, 2021. **24**(4): p. 374-388.

84. Vemuri, S., et al., *A questionnaire study on knowledge and awareness of metabolic syndrome and it’s components in undergraduate medical students at entry level.* International Journal of Research in Pharmaceutical Sciences, 2020. **11**(3): p. 3645-3656.

85. Vibhute, N.A., et al., *Dietary habits amongst medical students: An institution-based study.* J Family Med Prim Care, 2018. **7**(6): p. 1464-1466.

86. Vijayan, V., P. Panchu, and B. Bahuleyan, *Does lifestyle of medical students have a role in determining body mass index and body fat percentage?* Journal of Clinical and Diagnostic Research, 2018. **12**(10): p. CD01-CD04.

87. Wang, Y., *Effectiveness of personality-informed interpersonal group counseling intervention among obese students in medical colleges.* Chinese Journal of School Health, 2022. **43**(4): p. 553-556.

88. Yeasmin, R., et al., *Assessment of overweight, obesity and metabolic variables among students of a private medical college in Dhaka city.* Bangladesh Journal of Medical Science, 2015. **14**(1): p. 53-58.

89. Yousif, M.M., L.A. Kaddam, and H.S. Humeda, *Correlation between physical activity, eating behavior and obesity among Sudanese medical students Sudan.* BMC Nutr, 2019. **5**: p. 6.

90. Zaidi, T.H., et al., *Night eating syndrome among medical students and its correlation with depression in Karachi, Pakistan.* Romanian Journal of Neurology/ Revista Romana de Neurologie, 2020. **19**(3): p. 193-199.

91. Zaidi, U., et al., *BMI and psychological constructs: Comparative study of medical and non-medical female students of Riyadh, Saudi Arabia.* Cogent Psychology, 2019. **6**(1).

92. Purohit, G., T. Shah, and J.M. Harsoda, *Prevalence of Obesity in Medical students and its correlation with cardiovascular risk factors: Emergency Alarm for Today?* Kathmandu Univ Med J (KUMJ), 2015. **13**(52): p. 341-5.

93. Savić, S., L. Gavran, and G. Tešanović, *Assessment of physical activity and body weight among medical students in Banja Luka, Bosnia and Herzegovina.* Med Glas (Zenica), 2020. **17**(1): p. 188-193.

94. Shanshal, A.M., et al., *Evaluating Eating Disorders among Medical Students in Baghdad, Iraq.* Al-Rafidain Journal of Medical Sciences, 2022. **3**: p. 6-13.

95. Sharma, S., et al., *Utility of Consensus Statement in Assessment of Obesity: A Study among Undergraduate Medical Students from Rural Northwest India.* J Family Med Prim Care, 2013. **2**(3): p. 274-6.

96. Wiese, H.J., et al., *Obesity stigma reduction in medical students.* Int J Obes Relat Metab Disord, 1992. **16**(11): p. 859-68.

97. Şahin, H., et al., *Obesity prevalence and related factors among medical students in Kayseri.* Erciyes Tip Dergisi, 2015. **37**(2): p. 51-58.

98. Tina, J., et al., *Prevalence of overweight & obesity-associated risk factors amongst medical students in south india.* International Journal of Research in Pharmaceutical Sciences, 2020. **11**(Special Issue 2): p. 76-80.

99. Wójtowicz-Chomicz, K. and A. Borzecki, *Does obesity occur among students of medical university in lublin?* Family Medicine and Primary Care Review, 2011. **13**(2): p. 251-253.
